# Supplementary material for: Rapid Synergistic Biofilm Production of Pseudomonas and Candida on the Pulmonary Cell Surface and in Mice, a Possible Cause of Chronic Mixed Organismal Lung Lesions
Source: Int J Mol Sci. 2022 Aug 16;23(16):9202. doi: 10.3390/ijms23169202 (PMC9409386; doi:10.3390/ijms23169202)
Supplement: Supplementary file 1 [file ijms-23-09202-s001.zip › ijms-1850023-supplementary.pdf]

International Journal of
Molecular Sciences

MDPI

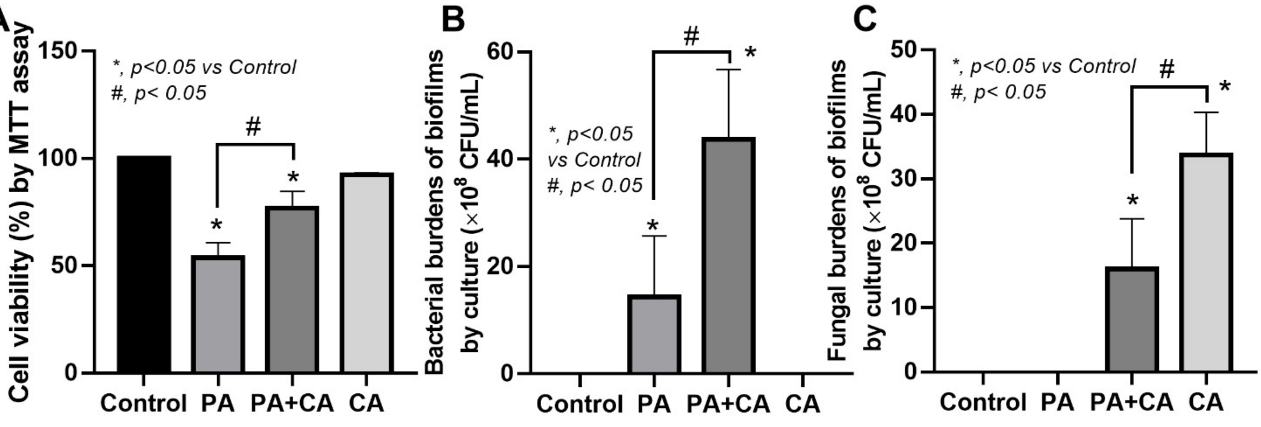

Figure S1. The cell viability (MTT assay) (**A**) of pulmonary cells (NCI-H292) after incubation by *Pseudomonas* alone (PA), *Candida* alone (CA), or *Pseudomonas* with *Candida* (PA+CA) or control media and the abundance of bacteria and fungi (**B**,**C**) in different conditions by culture are demonstrated (triplicated independent experiments were performed).

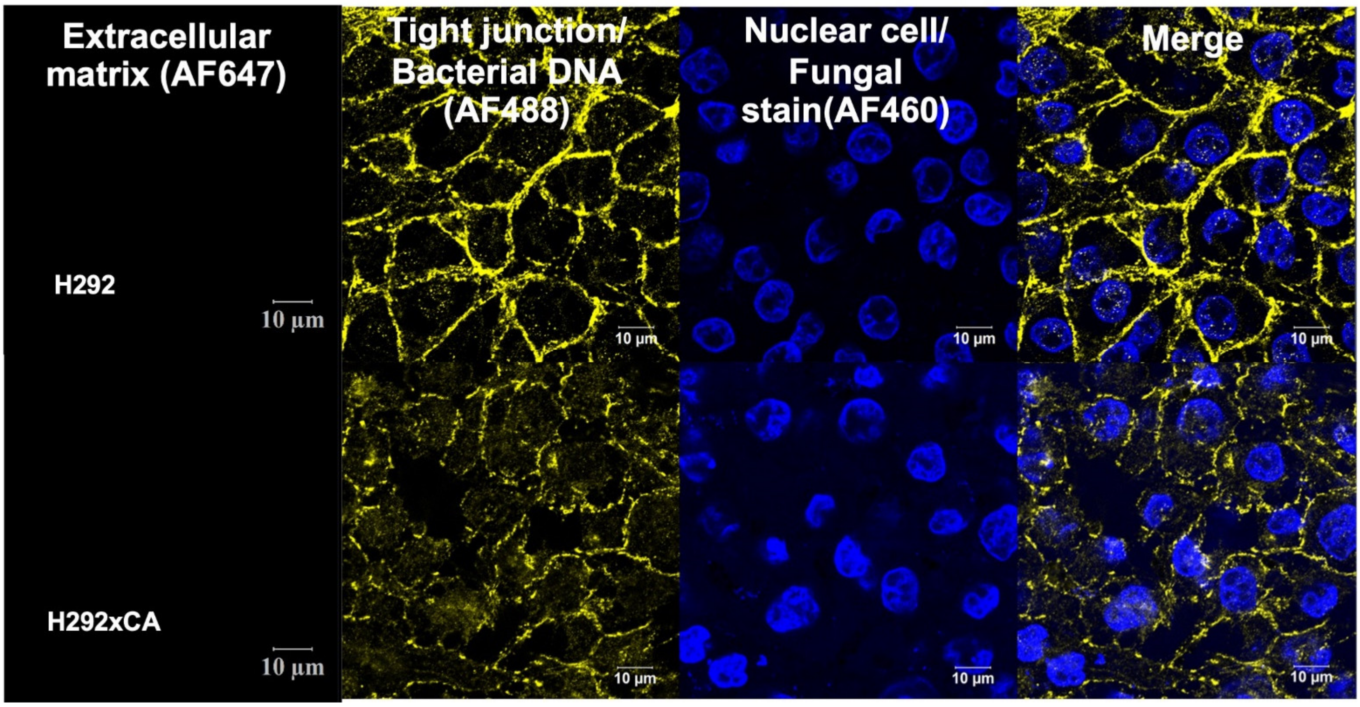

Figure S2. The fluorescent colors on the pulmonary epithelial cells (NCI-H292) with *Candida* (CA) or control after staining for extracellular matrix (AF467, red color), cell tight junction, and bacterial DNA (AF488, yellow color), nuclei of host cells and fungi (AF460, blue color) as indicated by the representative pictures demonstrate non-biofilms after *Candida* incubation.
